# c.761C>T Mutation Linked Hyper IgM Syndrome Presenting with Hypertransaminasemia and Arthritis

**DOI:** 10.4274/tjh.2014.0081

**Published:** 2014-12-05

**Authors:** Mehmet Halil Celiksoy, Stephan Borte, Aydan İkincioğulları, Meltem Ceyhan Bilgici, Filiz Karagöz, Ayhan Gazi Kalaycı, Alişan Yıldıran

**Affiliations:** 1 Ondokuz Mayıs University Faculty of Medicine, Department of Pediatric Allergy and Immunology, Samsun, Turkey; 2 Leipzig University Faculty of Medicine, Department of Clinical Immunolgy, Leipzig, Germany; 3 Ankara University Faculty of Medicine, Department of Pediatric Allergy and Immunology, Ankara, Turkey; 4 Ondokuz Mayıs University University Faculty of Medicine, Department of Pediatric Radiology, Samsun, Turkey; 5 Ondokuz Mayıs University Faculty of Medicine, Department of Pathology, Samsun, TurkeY; 6 Ondokuz Mayıs University Faculty of Medicine, Department of Pediatric Gastroenterology, Hepatology and Nutrition, Samsun, Turkey

## TO THE EDITOR

Hyperimmunoglobulin M syndrome (HIGM) is a primary immunodeficiency characterized with low IgG, IgA, and IgE and normal or elevated IgM levels due to a defect in class switching recombination [1]. In this report, we present a patient with CD40 ligand (CD40L) deficiency who had arthritis and hypertransaminasemia with no history of serious infection until 8 years of age.

**Figure 1:**
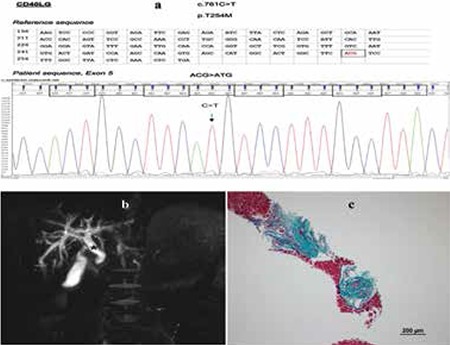
There is c.761C>T mutation in the CD40 ligand gene. b) MR cholangiography findings: dilated common choledochal (black star) and intrahepatic bile ducts, narrowing of the bile ducts, and irregularities at multiple levels (white arrows). c) Bile duct with increasing concentric connective tissue in one portal tract (trichrome stain, 10x).

An 8-year-old male patient presented because of a 2-year history of knee swelling and pain. The patient had no history of serious infection and he had been followed for asthma and elevated transaminase level by the Department of Pediatric Gastroenterology for the past 3 years. His parents were not relatives. Physical examination revealed the following: both knees had tenderness, swelling, redness, and limited range of motion. Other systems were normal. Laboratory analysis revealed the following results: Hb: 11.7 g/dL, Hct: 37.7%, MCV: 71.7 fL, leukocytes: 8.0x10^9^/L, platelets:405x10^9^/L, CRP: 18 mg/L, ESR: 25 mm/h, ALT: 10^3^ U/L (normal range: 0-55), AST: 63 U/L (5-40). The patient was negative for HBsAg, anti-HBs, anti-HCV, anti-CMV IgG, and CMV by polymerase chain reaction (PCR), while he was positive for anti-CMV IgM (252.7%, normal range: 90-100). Isohemagglutinins were 1/512 positive. The serum immunoglobulins were as follows: IgG: 1.27 g/L (5.5-17), IgA: 0.06 g/L (0.6-3.3), IgM: 5.09 g/L (0.6-2.7), IgE: 16 IU/mL. Examinations for arthritis did not reveal remarkable results and no pathogen could be isolated. Flow cytometry revealed CD40L deficiency, while analysis showed c.761C>T mutation in the same gene (Figure 1a). The patient was diagnosed with HIGM syndrome. A liver biopsy was done and magnetic resonance (MR) cholangiography was conducted for hypertransaminasemia. MR cholangiography and biopsy specimen findings were consistent with sclerosing cholangitis (Figures 1b and 1c). Informed consent was obtained.

In HIGM syndrome, clinical findings occur at early ages and the mean age of diagnosis is less than 1 year. Clinically, HIGM has similarities with recurring respiratory tract infections causing bronchiectasis and other humoral immune deficiencies presenting with sinus and ear infections [2]. Recurrent respiratory infections were absent in our case. Additionally, our patient was diagnosed with asthma. Recurrent respiratory infections may be confused with asthma attacks by physicians. Therefore, our patient could have been diagnosed late.

Although other markers were negative, anti-CMV IgM values were always high in this case. CMV-DNA PCR tests, applied at intervals, were also negative. In HIGM syndrome, the serum IgM level is normal or high but serum IgG and IgA levels are low, because of a defect in isotype class switching of B cells [3]. Furthermore, there is no response to protein antigens, though some IgM anti-polysaccharide antibodies, including isohemagglutinins, can be produced [2]. Therefore, our patient had CMV IgM probably due to CMV infection. However, he could not produce CMV IgG because of the nature of his disease.

Our patient was being monitored due to hypertransaminasemia in the gastroenterology outpatient clinic. Abdominal ultrasonography showed no cholangiopathy, but MR cholangiography findings were consistent with sclerosing cholangitis. Portal fibrosis was seen in only 1 of 4 vena portae samples in his liver biopsy. The most common characteristic finding of liver biopsy for sclerosing cholangitis is interlobular and septal fibrosis of bile ducts known as ‘onion skin’ [4]. Our findings of liver biopsy were not characteristic for sclerosing cholangitis. When clinical and radiological findings were evaluated together with liver biopsy results, the patient was diagnosed with sclerosing cholangitis in an initial state.

In conclusion, idiopathic arthritis and persistent hypertransaminasemia should also be considered in the differential diagnosis of primary immune deficiencies.

**Conflict of Interest Statement**

The authors of this paper have no conflicts of interest, including specific financial interests, relationships, and/or affiliations relevant to the subject matter or materials included.
